# Asthma treatment adherence among asthmatic patients in Yazd

**DOI:** 10.1186/1471-2458-12-S2-A39

**Published:** 2012-11-27

**Authors:** Azam Rahimi, Saperi Sulong, Nimetcan Mehmet Orhun, Hasanain Faisal Ghazi, Koffi Isidore Kouadio, Hidayatulfathi Othman

**Affiliations:** 1 United Nations University - International Institute for Global Health, Universiti Kebangsaan Malaysia Medical Centre, Jalan Yaacob Latiff, 56000 Kuala Lumpur, Malaysia; 2 Social Security Organization, Tehran, Iran; 3 Department of Health Information, Universiti Kebangsaan Malaysia Medical Centre, Jalan Yaacob Latiff, 56000 Kuala Lumpur, Malaysia; 4 Faculty of Health Sciences, Universiti Kebangsaan Malaysia, Jalan Raja Muda Abdul Aziz, 50300 Kuala Lumpur, Malaysia

## Background

Asthma affects an estimated 300 million people worldwide. Poor compliance with prescribed medication leads to increased morbidity and mortality. The number of asthma cases has more than doubled since 1980 and, nearly 3 million workdays are lost annually due to asthma exacerbations. Non-compliance with therapy is a major impediment to effective asthma management and can lead to failure of treatment. The purpose of the study was to identify the better predictor for adherence behaviors between knowledge of asthma, health beliefs, attitude toward the illness of asthma, and behavioral intention to adhere.

## Materials and methods

This cross sectional study was conducted among adult asthmatic patients in three private asthma clinics in Yazd city, Iran. Data was collected using face to face interview. During the interview a validated Questionnaire was used to assess patients’ knowledge about ICS,' positive and negative health beliefs and attitudes a about ICS, behavioral intention to comply with treatment, and compliance rate. A 4-item patient-report scale was used to measure medication adherence.

## Results

Response rate of participants 94%, adult (≥16 years old) with prescribed ICS reported were included (n=112). Analysis showed that majority of patients (55.5%) was non adherence to their prescribed ICS. Patients’ knowledge toward ICS did not have effect on medication adherence behavior, while patients with positive attitude toward ICS were better adherent with their medication. A simple linear regression identified intention to comply with treatment and positive attitude toward ICS as predictors for adherence behavior.

## Conclusions

The findings confirm a relationship between medication attitude and adherence among asthmatic patients. A better understanding of patients’ attitude and its impact on adherence may help clinicians counsel effectively to promote adherence.

